# Inhibition of the METTL3/m^6^A/miR-34a-5p axis suppresses trigeminovascular activation in nitroglycerin-induced migraine via the Wnt/β-catenin pathway

**DOI:** 10.1186/s10194-025-02144-7

**Published:** 2025-10-02

**Authors:** Hui Zhang, Minming Shao, Feng Zhang, Caiyan He, Jiabi Li, Shengdong He

**Affiliations:** 1 Department of Traditional Chinese Medicine, Affiliated Hospital of Guangdong Medical University, No.57 Renmin Avenue South, Xiashan District, Zhanjiang, 524000 Guangdong Province P. R. China; 2 Department of Respiratory and Critical Care Medicine, Xianhu Branch of the First Affiliated Hospital of Guangxi University of Chinese Medicine, No.327 Xianhu Avenue, Qingxiu District, Nanning, 530000 P. R. China

**Keywords:** METTL3, MiR-34a-5p, Wnt1/β-catenin pathway, Migraine

## Abstract

**Background:**

Neuroinflammation is a key driver of migraine pathogenesis, particularly by promoting neuronal sensitization. METTL3-mediated m^6^A methylation has emerged as a critical epigenetic regulator in neuroinflammatory responses. This study aims to investigate the role of METTL3 in migraine, focusing on its m^6^A-dependent regulatory mechanisms.

**Methods:**

A rat migraine model was induced via chronic intermittent nitroglycerin administration. Behavioral tests assessed migraine-like symptoms. Protein and RNA levels of METTL3, miR-34a-5p, and downstream targets were analyzed using Western blot, qPCR, ELISA, and immunofluorescence. The interaction among METTL3, miR-34a-5p, and Wnt1 was validated through Co-IP, RIP, and luciferase reporter assays.

**Results:**

METTL3 expression was significantly upregulated in the trigeminal ganglia of migraine rats. Knockdown of METTL3 reduced trigeminovascular system (TGVS) activation and alleviated migraine symptoms. Mechanistically, METTL3 enhanced miR-34a-5p expression via m^6^A modification, leading to suppression of the Wnt1/β-catenin pathway. Overexpression of miR-34a-5p further aggravated migraine-related responses by inhibiting Wnt1 signaling.

**Conclusion:**

METTL3 contributes to migraine pathogenesis through m^6^A-dependent upregulation of miR-34a-5p, which suppresses the Wnt1/β-catenin axis and promotes TGVS activation. Targeting this pathway may offer new therapeutic avenues for migraine.

**Supplementary Information:**

The online version contains supplementary material available at 10.1186/s10194-025-02144-7.

## Highlights

Depletion of METTL3 inhibits the activation of the trigeminovascular system, thereby alleviating migraine symptoms.

METTL3 upregulates miR-34a-5p by mediating m^6^A modification, thereby modulating the activation of the trigeminovascular system.

miR-34a-5p inhibits the Wnt1/β-catenin pathway, thereby modulating molecules related to the trigeminovascular system and exacerbating migraine.

**Supplementary Information:**

The online version contains supplementary material available at 10.1186/s10194-025-02144-7.

## Introduction

Migraine is a common and disabling primary headache disorder, impacting roughly 15% of people worldwide [1, 2]. It is characterized by recurrent, moderate-to-severe unilateral throbbing pain, often accompanied by photophobia, phonophobia, and nausea [1, 2]. Current migraine-specific therapeutics primarily consist of three major classes: triptans (5-HT1B/1D receptor agonists), gepants (small-molecule calcitonin gene-related peptide (CGRP) receptor antagonists), and CGRP monoclonal antibodies [3, 4]. While these pharmacological advancements have significantly improved migraine management, offering relief for many patients, several limitations persist. Clinical studies indicate that approximately 30–40% of patients show inadequate response to triptans, and the long-term safety profiles of newer CGRP-targeting therapies remain under investigation, particularly regarding potential cardiovascular and hepatic effects [5, 6]. These therapeutic challenges, along with the complex pathophysiology involving neurogenic inflammation, cortical spreading depression, and neuronal sensitization, highlight the urgent need to identify novel therapeutic targets.

Extensive studies on migraine have shown that neuroinflammatory responses, as well as the activation and sensitization of the trigeminovascular system (TGVS) play vital roles in the onset and development of migraines [7]. The TGVS is a central component of migraine pathophysiology [8, 9]. Its activation leads to the release of neuropeptides (such as CGRP and substance P), triggering vasodilation, neuroinflammation, and the transmission of pain signals, thereby inducing migraine attacks [10, 11]. In recent years, the regulation of the TGVS has become an important focus in the research of migraine treatment. For example, studies have found that baicalin (a flavonoid compound extracted from the Chinese herb Scutellaria baicalensis) can significantly alleviate pain behaviors in a rat migraine model induced by nitroglycerin (NTG) by inhibiting the activation of the trigeminal system [12]. Additionally, electroacupuncture therapy has also been proven to downregulate the expression of CGRP in the trigeminal ganglion and caudal trigeminal nucleus of migraine rats, thereby inhibiting neuroinflammation and vasodilation and effectively relieving migraine symptoms [13]. These findings suggest that inhibiting the activation of the TGVS is one of the key mechanisms for alleviating migraines.

There is growing evidence that neuronal dysfunction and hyperexcitability play a central role in migraine pathophysiology. Neurons in the TGVS act as key effectors that transmit and amplify nociceptive signals during migraine attacks, and their abnormal activation leads to sensitization and enhanced pain perception [14]. Recent studies have revealed that methyltransferase-like 3 (METTL3), a critical N6-methyladenosine (m^6^A) RNA methyltransferase, regulates neuronal functions by modulating mRNA stability and translation. Dysregulation of METTL3-mediated m^6^A modification in neurons has been linked to aberrant synaptic plasticity, neuronal excitability, and neuroinflammation, all of which are closely related to migraine mechanisms [15]. Furthermore, inhibition of METTL3 has been shown to alleviate neuronal sensitization and inflammatory pain in animal models [16]. Despite these findings, the precise role of METTL3 in migraine and its downstream molecular pathways remains largely unexplored.

MicroRNAs (miRNAs), a class of endogenous small non-coding single-stranded RNAs approximately 22 nucleotides in length, play crucial regulatory roles in the pathogenesis and development of migraine [17]. Our preliminary research findings have indicated that miR-34a-5p level is enhanced in rat models of migraine and positively correlates with CGRP levels [18]. In vitro studies further support miR-34a-5p as a potential target for migraine intervention [19]. Recent evidence suggests that m^6^A modification regulates miRNA maturation and influences target gene expression [20]. For instance, METTL3 promotes the maturation of miR-221/222 through mediating m^6^A methylation, leading to the subsequent downregulation of phosphatase and tensin homolog (PTEN) expression [21]. Silencing of fat mass and obesity-associated protein (FTO) enhanced m^6^A-dependent pri-miR-10a processing, facilitating miR-10 maturation and subsequent suppression of myotubularin-related protein 3 (MTMR3) expression, which ultimately contributed to glioma progression [22]. Notably, m^6^A modifications promote the recognition and processing of primary miRNAs (pri-miRNAs) by enhancing their binding to DiGeorge syndrome critical region gene 8 (DGCR8), a key component of the microprocessor complex along with Drosha [23]. DGCR8 binds to the stem-loop structure of pri-miRNAs and, in cooperation with Drosha, cleaves them into precursor miRNAs (pre-miRNAs), an essential step in miRNA biogenesis. METTL3-catalyzed m^6^A methylation has been shown to increase DGCR8’s affinity for specific pri-miRNAs, thereby facilitating their maturation into functional miRNAs. We predicted and identified potential binding sites between miR-34a-5p and the 3’UTR region of Wnt1 using the TargetScan database. Notably, Tanha et al. demonstrated that elevated levels of dickkopf-related protein 1 (DKK1), a potent inhibitor of the Wnt signaling pathway, exhibit a significant causal association with migraine pathogenesis [24]. This finding suggests that modulation of the Wnt/β-catenin axis may offer new therapeutic potential for migraine. However, whether METTL3 upregulates the expression of miR-34a-5p by mediating m^6^A methylation modification of pri-miR-34a, and subsequently influences the pathogenesis of migraine through targeting Wnt1, remains to be elucidated.

The objective of this study is to determine whether METTL3 facilitates the expression of miR-34a-5p through m^6^A methylation of pri-miR-34a, and whether this miRNA, by targeting Wnt1, suppresses the Wnt/β-catenin signaling pathway, thereby enhancing TGVS activation and contributing to migraine pathogenesis.

## Materials and methods

### Animals

Male Sprague-Dawley rats (205–240 g, 7 weeks old) were obtained from Hunan SJA Laboratory Animal Co., Ltd. (Changsha, China) and housed under specific pathogen-free (SPF) conditions. The rats were maintained on a 12-hour light/dark cycle, with controlled Humidity at 60± 5% and a temperature of 22 ± 2 °C. Food and water were provided ad libitum. All animal procedures were approved by Institutional Animal Care and Use Committee.

### Establishment of migraine models

The migraine model was established through the repeated administration of NTG following established protocols from prior research [25]. A stock solution of NTG (5.0 mg/mL) was diluted to 1 mg/mL using 0.9% saline for injection. Rats received intraperitoneal (i.p.) injections of the diluted NTG at a dosage of 10 mg/kg or an equivalent volume of 0.9% saline every other day over a nine-day period, resulting in a total of five injections.

Only male rats were used in this study to avoid the potential influence of hormonal fluctuations associated with the estrous cycle, which can affect pain perception, neuroinflammation, and behavioral outcomes. This design choice is consistent with previous research using the NTG-induced migraine model [26, 27].

Animals were randomly assigned to experimental groups using a random number table. All behavioral tests, sample collections, and data analyses were performed by researchers who did not know the group allocations to minimize bias and enhance reproducibility.

## Experimental design

### Experiment 1

To probe the function of METTL3 in the pathogenesis of migraine, animals were randomly assigned to four experimental groups (*n* = 6 per group): sham, NTG, NTG + Ad-sh-NC (adenovirus expressing negative control shRNA), and NTG + Ad-sh-METTL3 (adenovirus expressing shRNA targeting METTL3). The sham and NTG groups were administered equivalent volumes of 0.9% saline or NTG (10 mg/kg), respectively, every other day (on days 1, 3, 5, 7, and 9). Adenoviruses containing sh-NC or sh-METTL3 were obtained from Hanbio (Shanghai, China). For the NTG + Ad-sh-NC and NTG + Ad-sh-METTL3 groups, rats received intracerebroventricular injections with Ad-sh-NC (1 × 10⁹ PFU) or Ad-sh-METTL3 (1 × 10⁹ PFU), followed by NTG injection 30 min later. Adenoviruses were administered via intracerebroventricular injection to allow broad distribution within the nervous system. Adenoviral solutions were diluted in sterile phosphate-buffered saline (PBS) to a final volume of 10 µL. Rats were deeply anesthetized with 2% isoflurane and secured in a stereotaxic apparatus. A Hamilton syringe was used to slowly inject 10 µL of the viral suspension into the left lateral ventricle over 10 min (1 µL/min). To minimize reflux, the needle was left in place for an additional 5 min following injection. This injection procedure was repeated every other day throughout the modeling period (days 1, 3, 5, 7, and 9) to ensure sustained knockdown of METTL3. To facilitate repeated intracerebroventricular injections of Ad-shRNA vectors, rats underwent stereotaxic surgery for lateral ventricle cannula implantation (coordinates: −0.8 mm posterior to bregma, 1.5 mm lateral, 3.6 mm deep). The cannula was secured with dental cement, and animals were allowed to recover for 7 days prior to NTG or adenoviral injection. This setup enabled repeated and accurate delivery of Ad-sh-METTL3 or Ad-sh-NC vectors without re-surgery.

### Experiment 2

To investigate the role of miR-34a-5p in migraine pathogenesis, animals were randomly assigned to four experimental groups (*n* = 6 per group): sham, NTG, NTG + antagomir NC, and NTG + miR-34a-5p antagomir. The administration protocols for the sham and NTG groups were identical to those described in Experiment 1. In the NTG + antagomir NC and NTG + miR-34a-5p antagomir groups, chemically modified antagomir NC and miR-34a-5p antagomir were purchased from GenePharma (Shanghai, China). Following NTG injection 30 min, rats were deeply anesthetized with 2% isoflurane and secured in a stereotaxic apparatus. A total of 4 µL of PBS containing 1.4 nmol of miR-34a-5p antagomir or negative control antagomir (final concentration: 350 µM) was injected into the left lateral ventricle (coordinates: −0.8 mm posterior to bregma, 1.5 mm lateral, 3.6 mm deep) using a Hamilton microsyringe. The injection was performed at a constant rate of 0.8 µL/min, and the needle was held in place for an additional 5 min post-injection to prevent backflow. Like adenoviral vectors, antagomir administration were also performed every other day during the modeling period. The schematic diagram illustrating the modeling procedures for Experiments 1 and 2 is provided below.


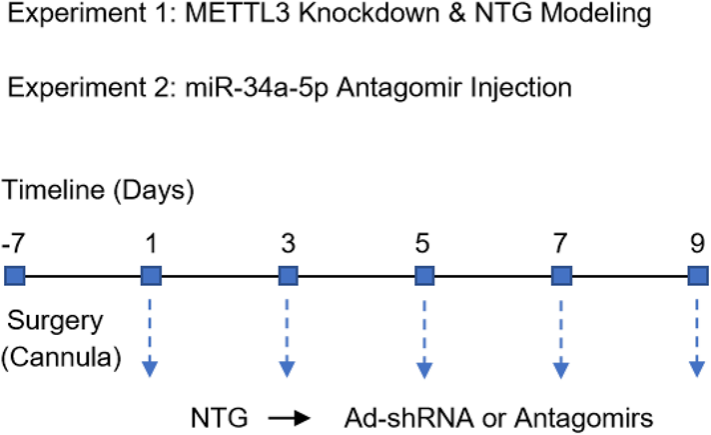


### Behavioral tests

All behavioral experiments were performed in a quiet, temperature-controlled environment by two investigators who were unaware of the treatment groups. To minimize potential confounding effects from surgical intervention (e.g., cannula implantation), a 7-day postoperative recovery period was allowed before the initiation of NTG modeling and any behavioral testing. This recovery duration ensured that inflammation or discomfort associated with surgery did not influence pain threshold assessments. Prior to the start of behavioral measurements, rats were habituated to the testing chamber for 2 h/day over 3 consecutive days to reduce novelty-induced stress. On testing days, rats were further acclimated for 30 min before each behavioral session. Baseline measurements of mechanical thresholds and thermal withdrawal latency were obtained 20 min prior to each NTG or saline injection. The same animals were used across all behavioral testing timepoints to allow within-subject comparisons.

### Mechanical allodynia

Mechanical sensitivity was evaluated using calibrated von Frey filaments following established protocols [25]. Rats were placed individually in clear cylindrical enclosures on an elevated metal mesh platform. Periorbital testing involved perpendicular application of filaments to the caudal eye region, with positive responses defined as head withdrawal or face scratching. For hind paw assessment, filaments were applied to the mid-plantar surface, and a positive response was defined as a sudden paw withdrawal, shaking, or licking. Mechanical withdrawal thresholds were determined using the up-down method as described by Chaplan et al. [28], which is based on the Dixon up-down paradigm. Testing began with a filament force of 2.0 g. If a positive response occurred, the next weaker filament was applied; if no response occurred, the next stronger filament was used. This process was repeated until six measurements were made after the first response change (from positive to negative or vice versa). The 50% paw withdrawal threshold was then calculated using the formula provided by Dixon and adapted by Chaplan et al. [28] for von Frey filaments. This method allows precise quantification of mechanical allodynia using a standardized algorithm.

### Thermal hyperalgesia

Thermal hyperalgesia was assessed using a modified method from Zhang et al. [25]. Briefly, the rat was placed in a plexiglass enclosure on a hot plate set to 55 °C, with a 30-second cut-off to prevent injury. The paw withdrawal latency, indicated by paw licking, raising, or jumping off, was recorded as the response to thermal hyperalgesia. The final withdrawal latency was calculated as the average of three tests with five-minute intervals.

### Trigeminal neuron cell culture and transfection

Rat trigeminal neuron cells used in this study were primary cultured neurons obtained from Procell Life Science & Technology Co., Ltd. (Catalog No. CP-R314, Wuhan, China). These neurons were isolated from the trigeminal ganglia of neonatal Sprague-Dawley rats using enzymatic digestion and differential adherence, and then cultured according to the manufacturer’s protocol. Cells were maintained in complete neuron medium (CM-R314) at 37 °C in a humidified atmosphere with 5% CO_2_. According to the supplier’s quality control data, these cells were ≥ 90% pure, as confirmed by immunofluorescence staining for β-Tubulin III, a neuronal marker. The cells were non-immortalized. These features ensured that the cells retained primary trigeminal neuron characteristics and were suitable for investigating migraine-related trigeminal neuronal responses in vitro. For transfection, neurons were transfected using Lipofectamine 3000 with: (1) METTL3/Wnt1 overexpression plasmids (oe-METTL3/oe-Wnt1) or negative control (oe-NC, Hanbio, Shanghai; 1 µg DNA + 2 µL P3000 Enhancer in Opti-MEM); or (2) miR-34a-5p agomir/antagomir (50 nM final concentration, GenePharma, Shanghai; 1:1 for miRNA: Lipofectamine ratio). Complexes were incubated (15 min) before 6-hour exposure, followed by complete medium replacement. Transfection efficiency was validated at 48 h.

### qRT-PCR

RNA isolation was carried out with TRIzol Reagent (Invitrogen), followed by reverse transcription utilizing the High-Capacity cDNA Reverse Transcription Kit (Applied Biosystems). qRT-PCR analysis was conducted on the LightCycler 480 II platform (Roche, Basel, Switzerland) with ChamQ Universal SYBR qPCR Master Mix (Vazyme, Nanjing, China). The internal reference gene GAPDH or U6 was employed for normalization, and quantification of gene level was calculated through the comparative threshold cycle (2^−ΔΔCt^) approach.

### Western blot assay

Cells or tissues were lysed in RIPA lysis buffer supplemented with protease and phosphatase inhibitors. Total protein concentration was determined using a BCA protein assay kit (Beyotime, China). Equal amounts of protein (30 µg per sample) were separated by SDS-PAGE and transferred onto PVDF membranes (Millipore, Billerica, CA, USA). Membranes were blocked with 5% skimmed milk in TBST for 1 h at room temperature, and then incubated overnight at 4 °C with the following primary antibodies: METTL3 (ab195352, 1:1000, Abcam), COX2 (ab179800, 1:1000, Abcam), CGRP (ab189786, 1:1000, Abcam), PACAP (sc-166180, 1:500, Santa Cruz Biotechnology), c-Fos (AF5354, 1:1000, Affinity Biosciences), Wnt1 (AF5315, 1:1000, Affinity Biosciences), β-catenin (AF6266, 1:1000, Affinity Biosciences), and GAPDH (AF7021, 1:10000, Affinity Biosciences). After washing, membranes were incubated with HRP-conjugated secondary antibodies for 1 h at room temperature, and the immunoreactive bands were visualized using enhanced chemiluminescence (ECL) reagents (Beyotime). Band intensities were quantified using ImageJ software (NIH), and target protein levels were normalized to GAPDH as an internal control. All Western blot experiments were performed in triplicate, and the relative protein expression was expressed as the ratio of target protein to GAPDH.

### Immunofluorescence assay

Frozen tissue slices were fixed with 4% paraformaldehyde for 20 min, permeabilized with 0.3% Triton X-100 for 15 min, and blocked with 5% bovine serum albumin (BSA) for 1 h at room temperature. The slices were then incubated overnight at 4 °C with primary antibodies against METTL3 (ab195352, 1:1000, Abcam) and c-Fos (AF5354, 1:200, Affinity Biosciences). After washing, slices were incubated with Alexa Fluor-conjugated secondary antibodies (S0018, 1:200, Affinity Biosciences) for 1 h at 37 °C in the dark, followed by nuclear staining with DAPI (Beyotime) for 10 min. Immunofluorescence images were captured using an Olympus fluorescence microscope (Japan) at 20× and 40× magnifications. For quantification, three randomly selected fields per section and three sections per animal were analyzed. The number of c-Fos–positive or METTL3–positive cells was manually counted using ImageJ software, and results were expressed as the average number of positive cells per field. All imaging parameters (exposure time, gain, etc.) were kept constant across all groups.

### ELISA

The levels of nitric oxide (NO), CGRP, and PACAP in serum or cell supernatant samples were measured using NO Colorimetric Assay Kit (E-BC-K135-M, Elabscience, Wuhan, China), Rat CGRP ELISA Kit (E-EL-R0135, Elabscience), and Rat PACAP ELISA Kit (ER0514, FineTest, Wuhan, China).

### Co-immunoprecipitation (Co-IP)

Following treatments, harvested cell lysates were processed with lysis buffer. The samples underwent antibody (anti-DGCR8: ab191875, 1:60, Abcam; anti-METTL3: ab195352, 1:50, Abcam; or normal IgG) incubation at 4 °C overnight, followed by incubation with protein A/G beads (sc-2003, Santa Cruz Biotechnology) at 4 °C for 2 h. Subsequently, immunocomplexes were washed with lysis buffer for 3 times and then subjected to Western blot analysis using anti-DGCR8 or anti-METTL3.

### RIP assay

UV-irradiated METTL3-overexpressing and control cells (254 nm, 400 mJ/cm²) were lysed in RIP buffer (Magna RIP Kit) and sonicated. Lysates were immunoprecipitated overnight at 4 °C with anti-DGCR8 or IgG control antibodies, followed by Proteinase K treatment. Co-precipitated RNAs were extracted via phenol/chloroform and analyzed by qRT-PCR using pri-miR-34a-specific primers.

For m^6^A analysis, DNase I-treated RNA was fragmented by sonication and incubated with m^6^A antibody-bound magnetic beads (ab195352, 1:50) in RIP buffer. After Proteinase K digestion (20 mg/mL, 42 °C, 90 min), RNA was extracted and quantified by qRT-PCR, normalized to input controls.

### Luciferase reporter assay

To validate the binding of miR-34a-5p to the 3’ untranslated region (3’UTR) of Wnt1, cells were seeded in 24-well plates at a density of 1 × 10^5^ cells/well and cultured overnight. Cells were co-transfected with 500 ng of psiCHECK2 vectors (Promega, Madison, WI, USA) containing either the wild-type (Wnt1-WT) or mutant (Wnt1-MUT) 3’UTR sequences and 50 nM miR-34a-5p agomir or negative control agomir, using Lipofectamine 3000 reagent (Thermo Fisher Scientific, USA) according to the manufacturer’s protocol. After 48 h of incubation, luciferase activity was measured using the Dual-Luciferase Reporter Assay System (Promega) on a luminometer. Renilla luciferase activity was normalized to Firefly luciferase activity to control for transfection efficiency. Each experiment was performed in triplicate and repeated at least three times independently. Data were expressed as relative luciferase activity (Renilla/Firefly ratio) normalized to the negative control group.

### Statistical analysis

All data are presented as mean ± standard deviation (SD). Statistical analyses were performed using GraphPad Prism (v10.2.3). Two-tailed Student’s t-tests were used for comparisons between two groups. For comparisons involving more than two groups, one-way analysis of variance (ANOVA) followed by Tukey’s post hoc test was applied. A p-value < 0.05 was considered statistically significant. Exact p-values and effect sizes are provided in the Supplementary Table 1.

## Results

### METTL3 was upregulated in migraine rats

**Fig. 1:**
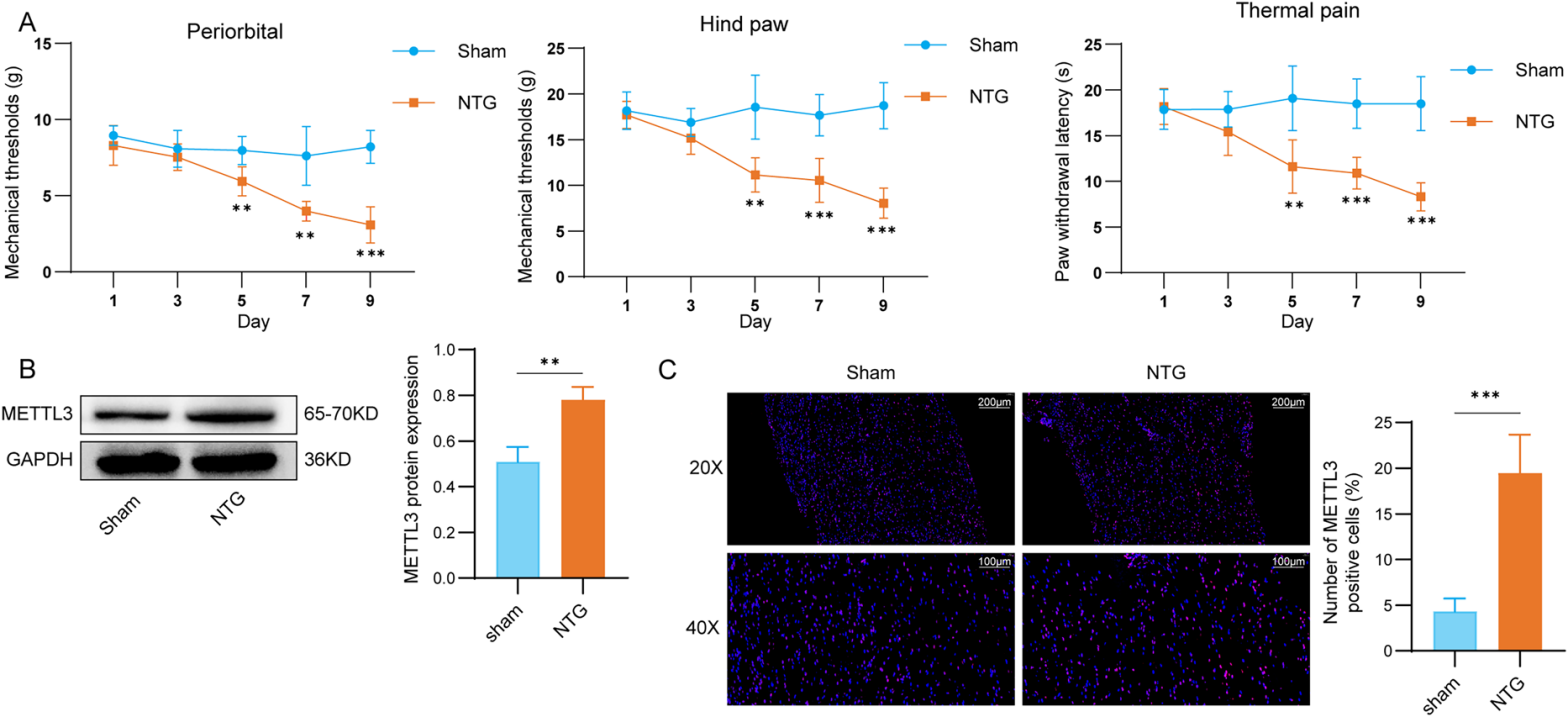
METTL3 was highly expressed in migraine rats. The experiment was divided into the following two groups: sham and NTG. (**A**) The basal mechanical pain threshold of periorbital and hind paw and the thermal pain threshold of the hind paw in each group. *n* = 6 per group. (**B**) Western blot assay was used to detect the expression of METTL3 in rat TG. *n* = 3 per group. (**C**) Immunofluorescence assay was used to detect the expression of METTL3 in rat TG (scale bar = 100 μm and 200 μm). *n* = 6 per group. Data are presented as mean ± SD. Statistical significance was determined using two-tailed unpaired t-test for two-group comparisons. **p < 0.01 and ***p < 0.001

In contrast to the saline-treated group, a progressive reduction in mechanical thresholds and latencies of the noxious heat response was observed following NTG administration (Fig. 1A). Furthermore, Western blot analysis demonstrated an upregulation of METTL3 protein expression in the trigeminal ganglia (TG) of NTG-triggered rats (Fig. 1B). Consistently, immunofluorescence analysis also revealed elevated expression of METTL3 in the TG of NTG-stimulated rats (Fig. 1C). These findings suggest that METTL3 is highly expressed in migraine and may contribute importantly to this disease progression.

### Depletion of METTL3 reduced TGVS activation and alleviated migraine symptoms

**Fig. 2:**
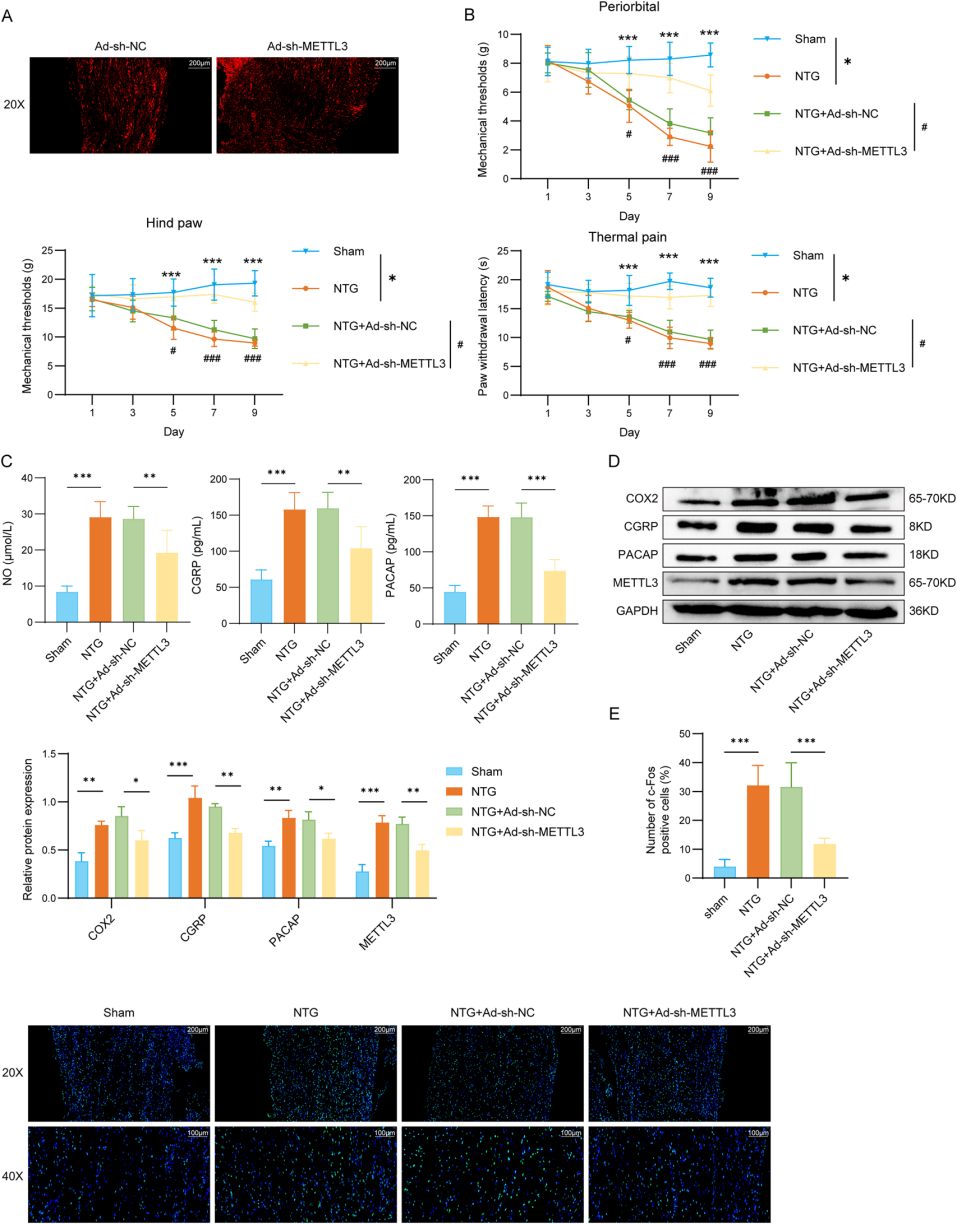
Depletion of METTL3 reduced TGVS activation and alleviated migraine. (**A**) Fluorescent images showing the expression of Ad-sh-METTL3 in TG (scale bar = 200 μm). The experiment was divided into four groups: sham, NTG, NTG + Ad-sh-NC, and NTG + Ad-sh-METTL3. (**B**) The basal mechanical pain threshold of the periorbital region and hind paw, as well as the thermal pain threshold of the hind paw, were measured in each group. (**C**) Serum levels of NO, CGRP, and PACAP were detected by ELISA in rats (*n* = 6 per group). (**D**) Western blot analysis was performed to assess COX2, CGRP, PACAP, and METTL3 protein levels in rat TG (*n* = 3 per group). (**E**) Immunofluorescence assay was conducted to detect c-Fos expression in rat TG (scale bar = 100 μm and 200 μm). *n* = 6 per group. Data are presented as mean ± SD. Statistical analysis was performed using one-way ANOVA followed by Tukey’s post hoc test. */#*p* < 0.05, **p < 0.01, and ***/###*p* < 0.001

As shown in fluorescent images in Fig. 2A, successful transduction and expression of Ad-sh-METTL3 in TG were confirmed by robust fluorescence signals. As illustrated in Fig. 2B, the mechanical thresholds and latencies to noxious heat in rats gradually decreased following NTG injection, and the effects of NTG were reversed after METTL3 depletion. Additionally, serum levels of NO, CGRP, and PACAP were significantly enhanced in rats subjected to NTG, while silencing METTL3 partially reversed these changes (reduced NO by 32.74%, reduced CGRP by 34.62%, reduced PACAP by 50.06%) (Fig. 2C). In the NTG group, the levels of COX2, CGRP, PACAP, and METTL3 in the TG were enhanced, whereas METTL3 silencing mitigated this rise (reduced COX2 by 29.78%, reduced CGRP by 28.71%, reduced PACAP by 24.31%) (Fig. 2D). Furthermore, c-Fos expression in the TG was elevated in NTG-triggered rats, while METTL3 knockdown effectively reduced its level by 62.48% (Fig. 2E). Overall, METTL3 knockdown attenuates TGVS activation and alleviated migraine symptoms.

### METTL3 upregulated miR-34a-5p by mediating m^6^A modification

**Fig. 3:**
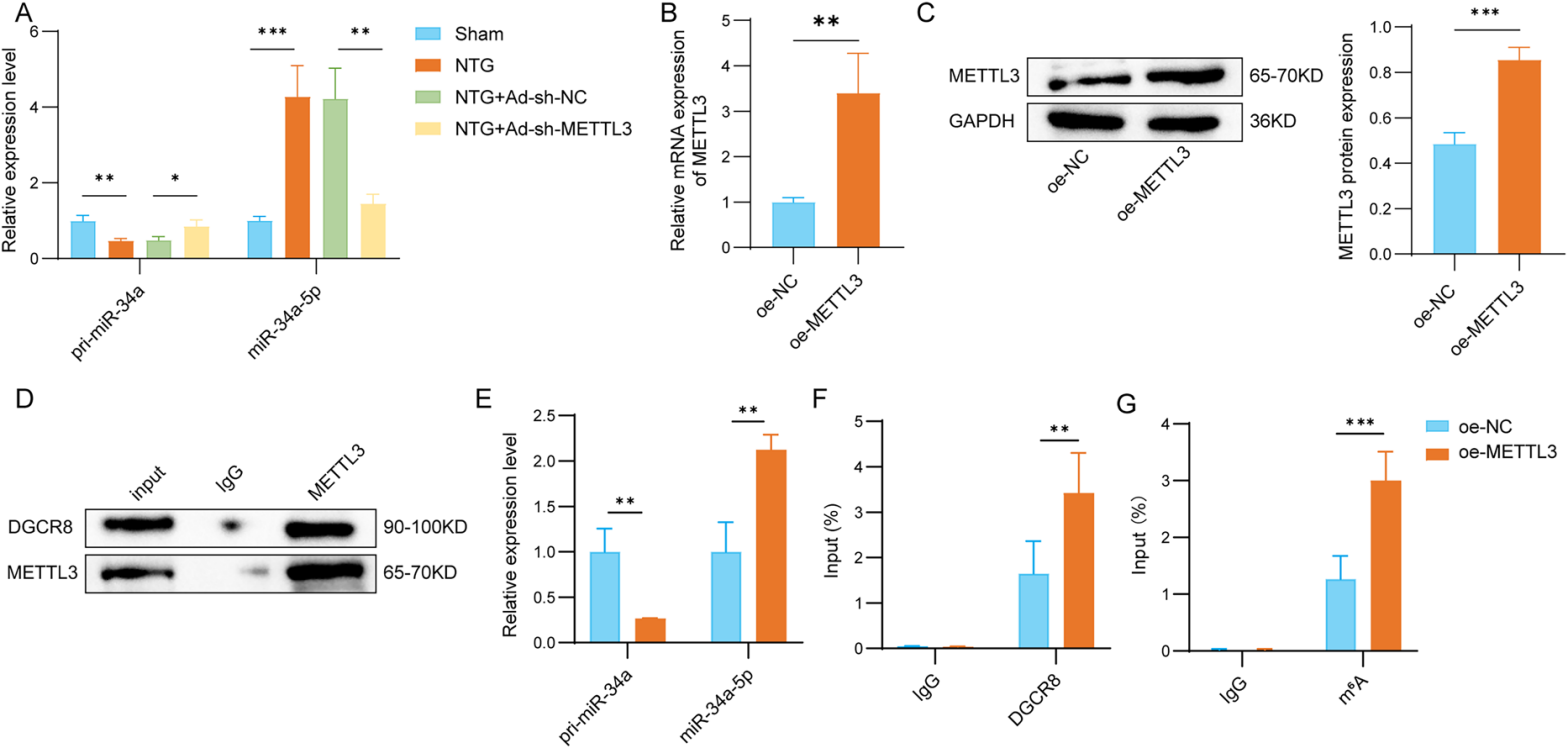
METTL3 upregulated miR-34a-5p by mediating m^6^A modification. (**A**) qRT-PCR assay was used to measure pri-miR-34a and miR-34a-5p mRNA levels in sham, NTG, NTG + Ad-sh-NC, and NTG + Ad-sh-METTL3 groups. (**B** and **C**) The mRNA and protein levels of METTL3 were measured by qRT-PCR and Western blot assays in METTL3-overexpressed rat trigeminal neurons. (**D**) Co-IP assay detected the relationship between METTL3 and DGCR8. (**E**) The levels of pri-miR-34a and miR-34a-5p was measured by qRT-PCR assay in METTL3-overexpressed rat trigeminal neurons. (**F**) The relationship between METTL3 and DGCR8 was confirmed by RIP assay in METTL3-overexpressed rat trigeminal neurons. (**G**) The enrichment of pri-miR-34a on m^6^A was confirmed by RIP assay. *n* = 3 for all panels. Data are presented as mean ± SD. One-way ANOVA with Tukey’s post hoc test or two-tailed unpaired t-test was used for statistical analysis. *p < 0.05, **p < 0.01, and ***p < 0.001

It has been established that miR-34a-5p is a crucial target for migraine treatment [17, 29]. Building on this foundation, we quantified the expression profiles of both pri-miR-34a and miR-34a-5p in the TG of rats. As illustrated in Fig. 3A, the depletion of METTL3 significantly inhibited the downregulation of pri-miR-34a and the upregulation of miR-34a-5p observed in the TG of NTG-treated rats. Subsequently, we overexpressed METTL3 and confirmed the transfection efficiency, as shown in Fig. 3B and C. Additionally, Co-IP results demonstrated that METTL3 interacts with DGCR8 (Fig. 3D). Notably, the addition of METTL3 distinctly downregulated pri-miR-34a and upregulated miR-34a-5p (Fig. 3E). The RIP assay indicated that the addition of METTL3 enhanced the binding of DGCR8 to pri-miR-34a (Fig. 3F). Furthermore, the overexpression of METTL3 facilitated the m^6^A modification of pri-miR-34a (Fig. 3G). In summary, METTL3 facilitates the recognition of pri-miR-34a by DGCR8 through m^6^A modification, thereby promoting its processing into mature miR-34a-5p.

### METTL3 modulated the levels of TGVS-related molecules through miR-34a-5p

**Fig. 4:**
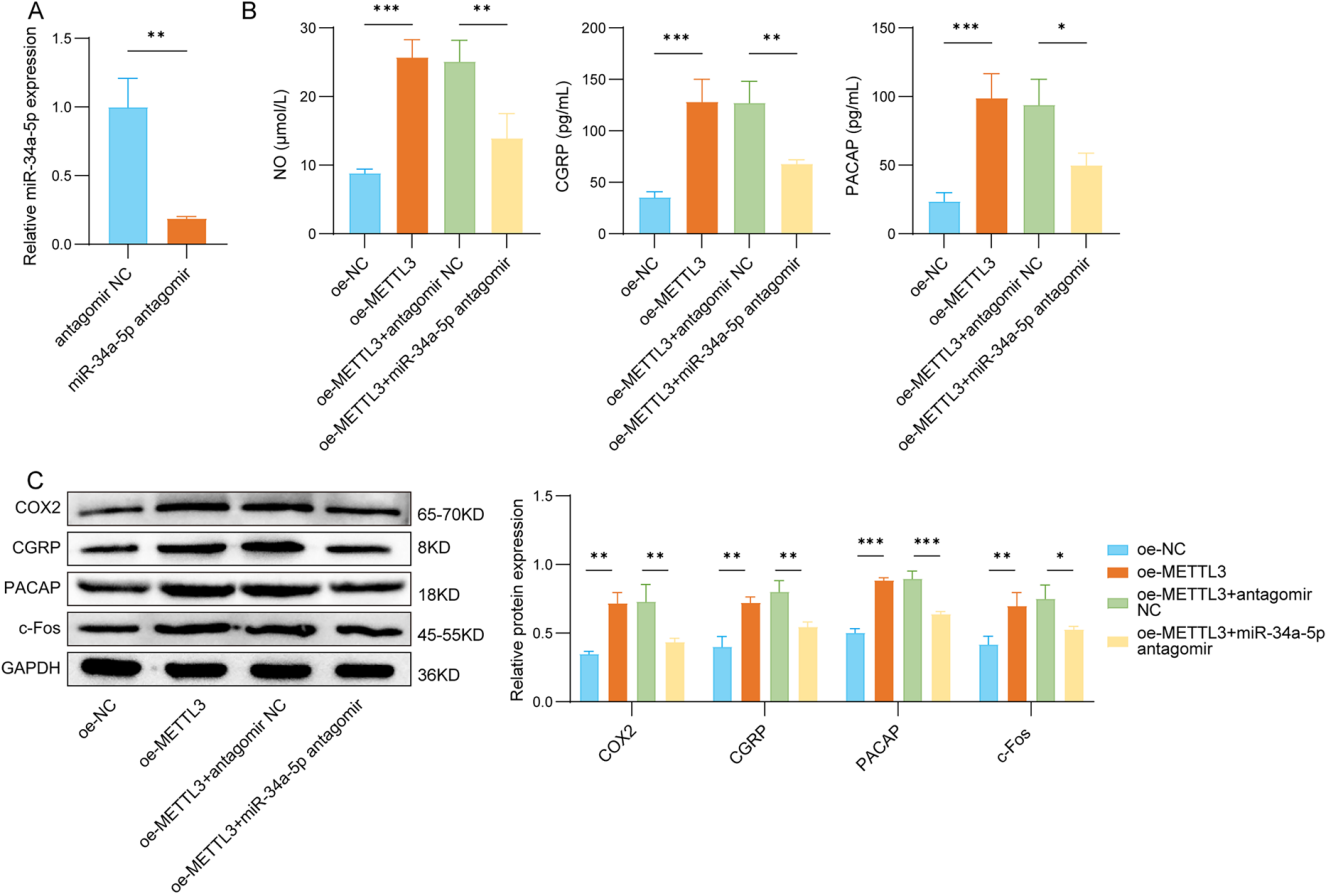
METTL3 modulated the levels of TGVS-related molecules through miR-34a-5p. (**A**) The expression of miR-34a-5p was detected by qRT-PCR in rat trigeminal neurons transfected with antagomir NC or miR-34a-5p antagomir. The following experiments are divided into the following groups: oe-NC, oe-METTL3, oe-METTL3 + antagomir NC, and oe-METTL3 + miR-34a-5p antagomir. (**B**) The levels of NO, CGRP, and PACAP were detected by ELISA assay in rat trigeminal neurons. (**C**) Western blot analysis of COX2, CGRP, PACAP, c-Fos protein levels in rat trigeminal neurons. *n* = 3 for all panels. Data are shown as mean ± SD. One-way ANOVA with Tukey’s post hoc test or two-tailed unpaired t-test was used for statistical analysis. *p < 0.05, **p < 0.01, and ***p < 0.001

Building on our findings that METTL3 promotes miR-34a-5p maturation via m^6^A-dependent processing (Fig. 3), we next examined whether the pro-migraine effects of METTL3 are functionally dependent on miR-34a-5p. We first inhibited miR-34a-5p in rat trigeminal neurons (Fig. 4A). As illustrated in Fig. 4B, the addition of METTL3 enhanced the production of NO, CGRP, and PACAP in rat TG neurons, whereas the inhibition of miR-34a-5p partially mitigated these effects. Furthermore, METTL3 markedly upregulated the protein levels of COX2, CGRP, PACAP, and c-Fos, whereas the inhibition of miR-34a-5p resulted in a partial downregulation of these proteins (Fig. 4C). Our findings expound that METTL3 facilitates the activation of the TGVS through the upregulation of miR-34a-5p.

### METTL3 inactivated the Wnt1/β-catenin pathway through miR-34a-5p

**Fig. 5:**
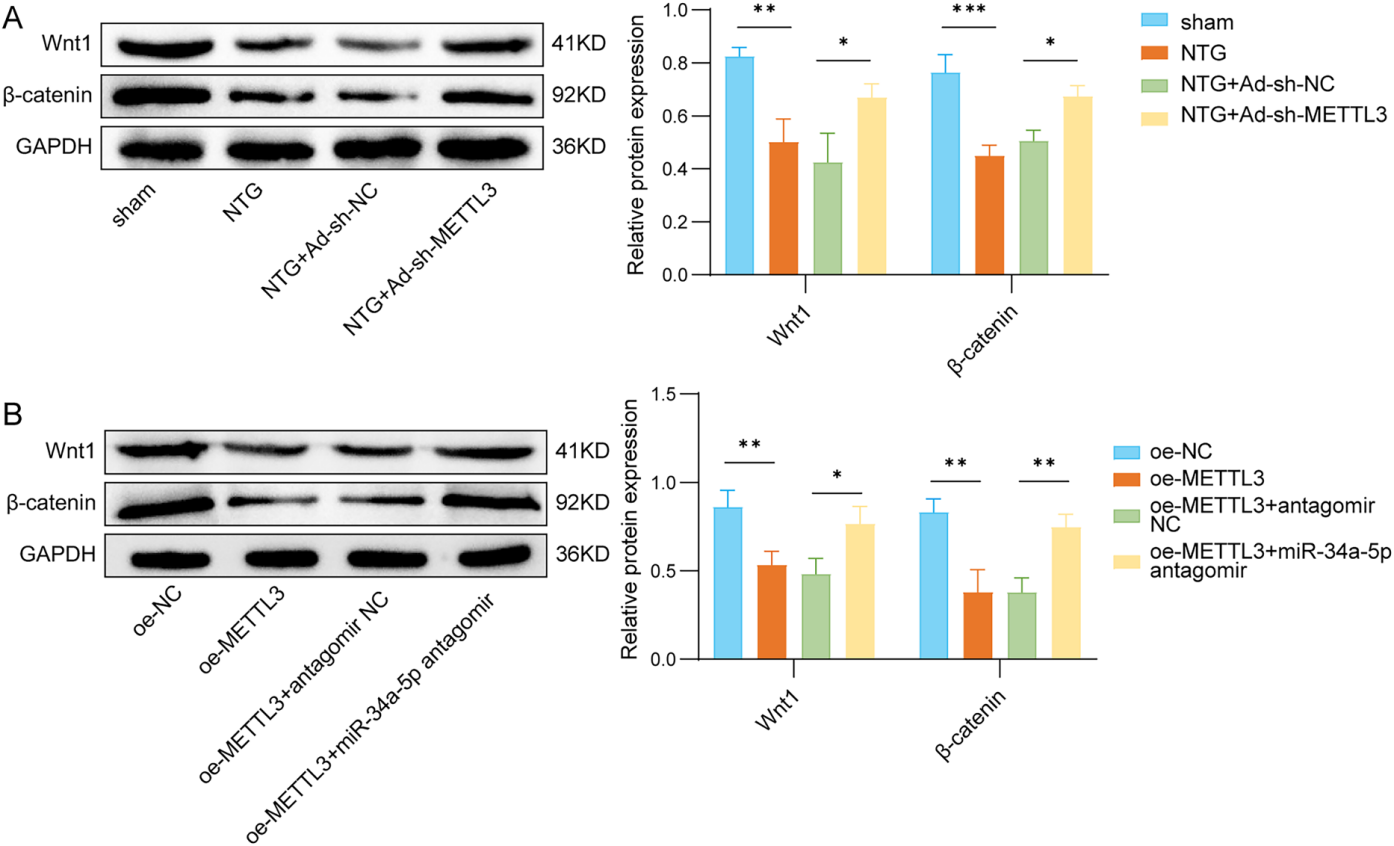
METTL3 inactivated the Wnt1/β-catenin pathway through miR-34a-5p. (**A**) Wnt1 and β-catenin protein levels were measured in rat TG in sham, NTG, NTG + Ad-sh-NC, and NTG + Ad-sh-METTL3 groups. (**B**) Wnt1 and β-catenin protein levels were measured in rat trigeminal neurons in oe-NC, oe-METTL3, oe-METTL3 + antagomir NC, and oe-METTL3 + miR-34a-5p antagomir groups. *n* = 3 for both panels. Data are expressed as mean ± SD. Statistical analysis by one-way ANOVA followed by Tukey’s post hoc test. *p < 0.05, **p < 0.01, and ***p < 0.001

Previous works have indicated that activation of the Wnt/β-catenin axis in the brain may play a role in alleviating migraine [24]. We assessed the levels of Wnt1 and β-catenin proteins in rat TG (Fig. 5A) and rat trigeminal neuron cells (Fig. 5B) using Western blot analysis. In the TG of rats treated with NTG, the expression of Wnt1 and β-catenin was significantly downregulated. Furthermore, the knockdown of METTL3 partially counteracted the effects induced by NTG (Fig. 5A). The addition of METTL3 significantly reduced the protein levels of Wnt1 and β-catenin in trigeminal neuronal cells, while silencing miR-34a-5p restored the expression of Wnt1 and β-catenin (Fig. 5B). These findings suggest that METTL3 inhibits activation of the Wnt1/β-catenin axis through miR-34a-5p.

### miR-34a-5p inhibited the activation of the Wnt1/β-catenin pathway

**Fig. 6:**
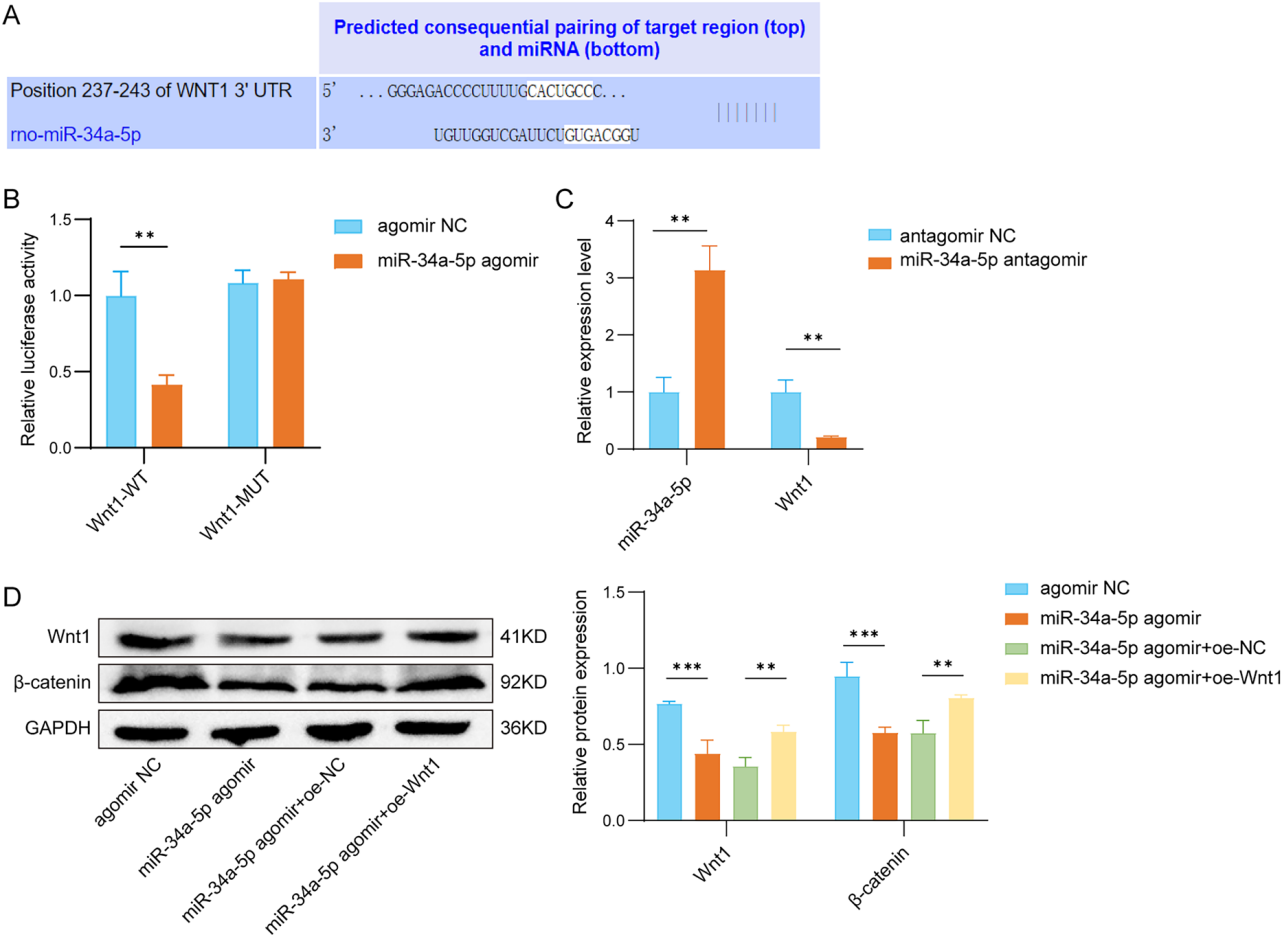
METTL3 inhibited the activation of the Wnt1/β-catenin pathway. (**A**) TargetScan was used to predict the binding site of miR-34a-5p to Wnt1. (**B**) Luciferase assay was performed to verify the binding of miR-34a-5p to Wnt1. (**C**) The expression of miR-34a-5p and Wnt1 was detected by qRT-PCR in rat trigeminal neurons transfected with antagomir NC or miR-34a-5p antagomir. The following experiments are divided into the following groups: antagomir NC, miR-34a-5p antagomir, miR-34a-5p antagomir + oe-NC, and miR-34a-5p antagomir + oe-Wnt1. (**D**) Wnt1 and β-catenin protein levels were measured by Western blot assay. *n* = 3. Data are shown as mean ± SD. Statistical significance determined by one-way ANOVA with Tukey’s post hoc test or two-tailed unpaired t-test. **p < 0.01 and ***p < 0.001

Using TargetScan, miR-34a-5p was predicted to have binding sites with Wnt1 (Fig. 6A). Besides, the addition of miR-34a-5p strikingly declined the luciferase activity of Wnt1-WT, while it had no impact on the luciferase activity of Wnt1-MUT (Fig. 6B). Besides, the overexpression of miR-34a-5p declined the mRNA levels of Wnt1 (Fig. 6C). Furthermore, the addition of miR-34a-5p led to a substantial decrease in the levels of Wnt1 and β-catenin proteins, whereas Wnt1 overexpression counteracted the influence of miR-34a-5p on the expression of these proteins (Fig. 6D). In summary, METTL3 can inhibit activation of the Wnt1/β-catenin axis by upregulating miR-34a-5p.

### miR-34a-5p modulated the levels of TGVS-related molecules through the Wnt1/β-catenin pathway

**Fig. 7:**
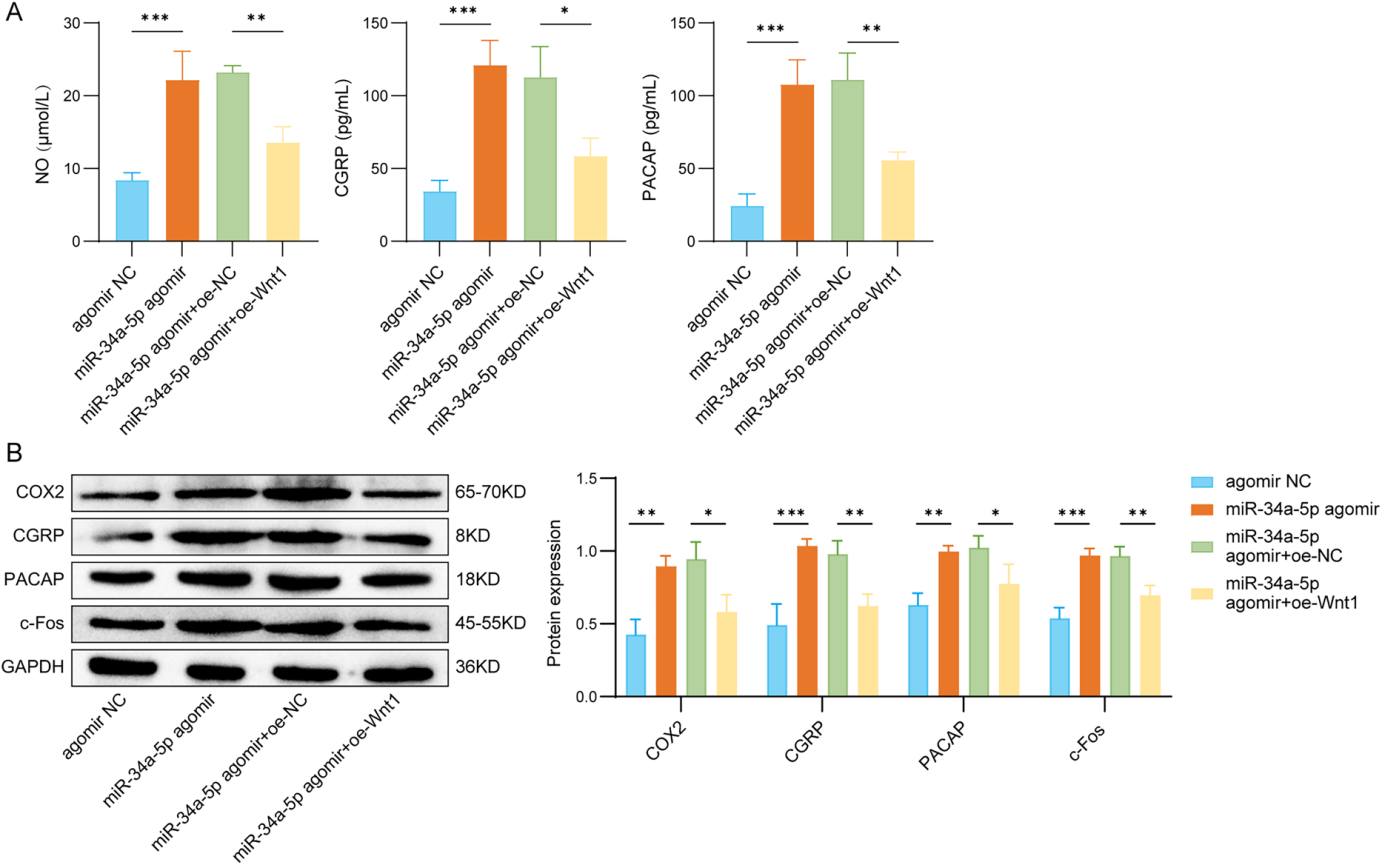
miR-34a-5p modulated the levels of TGVS-related molecules through the Wnt1/β-catenin pathway. The experiment was divided into the following four groups: antagomir NC, miR-34a-5p antagomir, miR-34a-5p antagomir + oe-NC, and miR-34a-5p antagomir + oe-Wnt1. (**A**) The levels of NO, CGRP, and PACAP were detected by ELISA assay. (**B**) Western blot analysis of COX2, CGRP, PACAP, and c-Fos protein levels in rat trigeminal neurons. *n* = 3 per group. Data are shown as mean ± SD. Statistical analysis by one-way ANOVA with Tukey’s post hoc test. *p < 0.05, **p < 0.01, and ***p < 0.001

The addition of miR-34a-5p markedly enhanced the levels of NO, CGRP, and PACAP, while addition of Wnt1 abolihsed the effects of miR-34a-5p (Fig. 7A). Furthermore, the overexpression of Wnt1 counteracted the upregulatory effects of miR-34a-5p on COX2, CGRP, PACAP, and c-Fos protein levels (Fig. 7B). In general, miR-34a-5p may regulate the activation of the TGVS through the Wnt1/β-catenin axis.

### miR-34a-5p aggravated migraine by regulating the Wnt1/β-catenin pathway

**Fig. 8:**
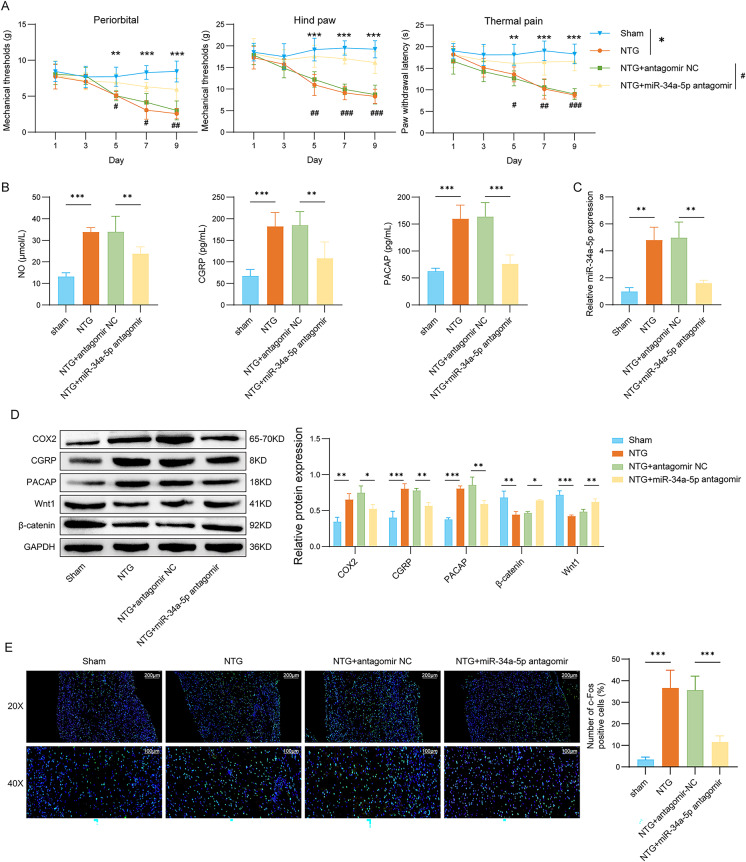
miR-34a-5p aggravated migraine by regulating the Wnt1/β-catenin pathway. The experiment was divided into the following four groups: sham, NTG, NTG + antagomir NC, and NTG + miR-34a-5p antagomir. (**A**) The basal mechanical pain threshold of periorbital and hind paw and the thermal pain threshold of the hind paw in each group. (**B**) The serum levels of NO, CGRP, and PACAP were detected by ELISA assay. *n* = 6 per group. (**C**) The expression of miR-34a-5p was measured by qRT-PCR. (**D**) COX2, CGRP, PACAP, Wnt1, and β-catenin protein levels in the TG of rats were detected by Western blot assay. *n* = 3 per group. (**E**) Immunofluorescence assay was used to detect the expression of METTL3 in rat TG (scale bar = 100 μm and 200 μm). *n* = 6 per group. Data are shown as mean ± SD. One-way ANOVA with Tukey’s post hoc test was used. */#*p* < 0.05, **/##*p* < 0.01, and ***/###*p* < 0.001

As shown in Fig. 8A, following NTG injection, the mechanical thresholds and latencies to noxious heat in rats progressively declined, an effect that was partially reversed upon the inhibition of miR-34a-5p. Moreover, knockdown of miR-34a-5p significantly reduced the elevated levels of NO, CGRP, and PACAP in the serum of NTG-treated rats (Fig. 8B). miR-34a-5p was significantly upregulated in the TG of rats treated with NTG, and this upregulation was reversed by miR-34a-5p silencing (Fig. 8C). miR-34a-5p inhibition significantly suppressed NTG-induced expression of COX2, CGRP, and PACAP while restoring the NTG-inhibited level of Wnt1 and β-catenin in the rat TG (Fig. 8D). Furthermore, the inhibition of miR-34a-5p led to a significant decrease in c-Fos levels in the TG of NTG-treated rats (Fig. 8E). These findings indicate that inhibiting miR-34a-5p can activate the Wnt1/β-catenin pathway and mitigate migraine symptoms mediated by the activation of TGVS.

## Discussion

Migraine is a complex neurovascular disorder characterized by excessive activation of the TGVS and neuronal sensitization [30, 31]. Emerging evidence suggests that epigenetic modifications, particularly m^6^A RNA methylation, are implicated in neurological diseases [32]. Our study demonstrates that METTL3, a key m^6^A methyltransferase, is significantly upregulated in migraine and contributes to disease progression by promoting the maturation of miR-34a-5p and regulating the Wnt1/β-catenin pathway.

The elevated expression of METTL3 in TG of migraine rats aligns with the established role of TGVS hyperactivity in migraine. TGVS activation, characterized by CGRP release, neurogenic inflammation, and neuronal sensitization, is a hallmark of migraine pathophysiology [33, 34]. Our finding that the depletion of METTL3 suppresses TGVS activation (e.g., reduced the expression levels of COX2, CGRP, PACAP, and c-Fos) suggests its critical role in sustaining trigeminal nociceptive signaling. m^6^A RNA modification modulates pain-related gene expressions [35, 36]. Previous studies have demonstrated that METTL3 contributes to hyperalgesia and neuroinflammation by regulating key inflammatory mediators and pain-associated pathways [37, 38]. While previous studies have highlighted the pro-inflammatory role of METTL3 in microglia, contributing to neuroinflammation and neuronal injury [39], our study focuses specifically on the role of METTL3 in trigeminal neurons. This distinction is critical, as the cell-type-specific function of METTL3 may vary substantially. The effects observed in our study—such as increased expression of CGRP, PACAP, and NO production—were derived from neuron-enriched cultures and TG, which suggest that METTL3 also exerts direct pro-nociceptive effects in neurons. The cell-autonomous action of METTL3 in neurons, independent of microglia, supports a distinct mechanistic axis in migraine pathophysiology. Further studies using in situ hybridization or single-cell RNA-seq would be valuable to dissect the relative contribution of METTL3 in neurons versus glial cells under migraine conditions. In line with this, our study provides new evidence linking METTL3 to migraine pathophysiology, highlighting its role in TGVS activation and pain sensitization. These findings suggest that METTL3 could serve as a potential target for migraine treatment by modulating neurogenic inflammation and trigeminal nociceptive signaling.

miR-34a-5p has been implicated in the pathogenesis of migraine, particularly through its involvement in neuroinflammation and pain sensitization [40]. Studies have shown that miR-34a-5p regulates neuronal excitability [41] and inflammatory responses [19], both of which contribute to the activation of the TGVS, a key driver of migraine attacks. Elevated miR-34a-5p levels have been associated with increased expression of pain-related molecules, further exacerbating migraine symptoms [40]. However, the upstream regulatory mechanisms governing miR-34a-5p expression in migraine remain largely unexplored. Recent research has highlighted the role of m^6^A modification in miRNA biogenesis, where m^6^A methylation facilitates the processing of pri-miRNAs into mature functional miRNAs [42, 43]. METTL3, a core m^6^A methyltransferase, catalyzes m^6^A modification on pri-miRNAs, thereby promoting their recognition and processing by the microprocessor complex [44]. Specifically, m^6^A modifications enhance the affinity of pri-miRNAs for DGCR8, a key component of the Drosha-DGCR8 complex, which cleaves pri-miRNAs into precursor miRNAs (pre-miRNAs), ultimately leading to the production of mature miRNAs [45]. In this study, we found that METTL3 promotes miR-34a-5p maturation through m^6^A-dependent pri-miRNA processing. Our results demonstrate that METTL3 facilitates the recognition of pri-miR-34a by DGCR8 via m^6^A modification, thereby enhancing its processing into mature miR-34a-5p. This finding establishes METTL3 as an upstream epigenetic regulator of miR-34a-5p in migraine, suggesting that m^6^A modifications play a crucial role in fine-tuning miRNA expression in the trigeminal system. Besides, we also found that METTL3 facilitates the activation of the TGVS through the upregulation of miR-34a-5p. To contextualize the role of miR-34a-5p in migraine, it is important to note that other miRNAs, such as miR-155 and miR-181a, have also been implicated in neuroinflammatory mechanisms underlying migraine. miR-155 and miR-181a are known to promote microglial activation and the production of pro-inflammatory cytokines [46–48]. In contrast, miR-34a-5p appears to act primarily in neurons. This neuron-centric mechanism distinguishes miR-34a-5p from glia-related miRNAs and suggests that it contributes to migraine pathophysiology through a distinct cellular and molecular axis.

The Wnt/β-catenin pathway plays a vital role in neuroinflammation and pain modulation [49]. Works have shown that Wnt signaling contributes to synaptic plasticity, neuronal survival, and inflammatory response regulation, all of which are critical factors in the pathogenesis of chronic pain, including migraine [50, 51]. In particular, the inhibition of Wnt/β-catenin signaling has been associated with increased neuronal excitability and pain hypersensitivity, suggesting that dysregulation of this pathway may exacerbate migraine symptoms [52, 53]. Our study reveals that miR-34a-5p was predicted to have binding sites with Wnt1, suggesting a direct post-transcriptional regulation of Wnt1 expression. This binding may lead to a reduction in the expression of Wnt1, which in turn inhibites β-catenin activation and its downstream signaling. The downregulation of Wnt/β-catenin signaling by miR-34a-5p likely contributes to the observed modulation of TGVS-related molecules. As expected, our results indicated that miR-34a-5p regulates the expression of TGVS-related molecules through the Wnt1/β-catenin pathway. In addition, silencing of miR-34a-5p can activate Wnt1/β-catenin and reduce migraine mediated by the TGVS activation. Besides, the regulatory influence of METTL3 on the Wnt/β-catenin pathway has been confirmed by several studies [54, 55]. Here, we also indicated that METTL3 regulates the Wnt1/β-catenin axis through miR-34a-5p.

Several limitations should be acknowledged. First, although we focused on male rats to avoid hormonal variability, estrogen is known to modulate the expression of migraine-related mediators such as CGRP [56]. Given the higher prevalence and severity of migraine in women [57, 58], it is critical to explore the role of METTL3 in female models and assess potential sex-specific regulatory mechanisms. Estrogen may influence the m^6^A epitranscriptomic landscape or interact with miRNA pathways, and future studies should investigate how hormonal status alters the METTL3/miR-34a-5p/Wnt1-β-catenin axis. While our study highlights the METTL3/miR-34a-5p/Wnt1 signaling axis as a key contributor to TGVS activation in migraine, we acknowledge that METTL3 may exert additional effects through alternative mechanisms. For instance, METTL3 has been reported to influence gene expression independent of m^6^A modification by interacting with transcriptional regulators or RNA-binding proteins [59, 60]. It is therefore possible that METTL3 modulates TGVS-related molecules through m^6^A-independent pathways that were not explored in this study. Moreover, other signaling cascades such as the PI3K/AKT, MAPK, or NF-κB pathways—previously linked to migraine and neuroinflammation—may also be regulated by METTL3 either directly or indirectly. Although our data support a model where METTL3 acts primarily through miR-34a-5p and the Wnt1/β-catenin axis, further investigation is warranted to determine whether additional parallel or converging mechanisms are involved. Future studies using METTL3 catalytic mutants, RNA immunoprecipitation followed by sequencing (RIP-seq), or transcriptome-wide m^6^A profiling (MeRIP-seq) will be essential to distinguish m^6^A-dependent from m^6^A-independent functions of METTL3 in migraine. Currently, there are no published clinical data showing changes in METTL3 or miR-34a-5p levels in the blood or trigeminal system of human migraine patients. Our study is limited to animal models and in vitro experiments. However, we agree that clinical validation is essential. Future studies will aim to examine METTL3 and miR-34a-5p expression in blood or cerebrospinal fluid from migraine patients, especially during acute attacks. Regarding the relationship with cortical spreading depression (CSD), while our study did not directly investigate CSD, it is known that CSD can activate the trigeminovascular system and initiate migraine pain. Therefore, it is plausible that CSD-induced TGVS activation may also involve METTL3-mediated mechanisms, especially in neurons. Further studies are needed to explore whether METTL3 expression is altered in CSD models and how it may intersect with CSD-triggered signaling pathways.

## Conclusion

**Fig. 9:**
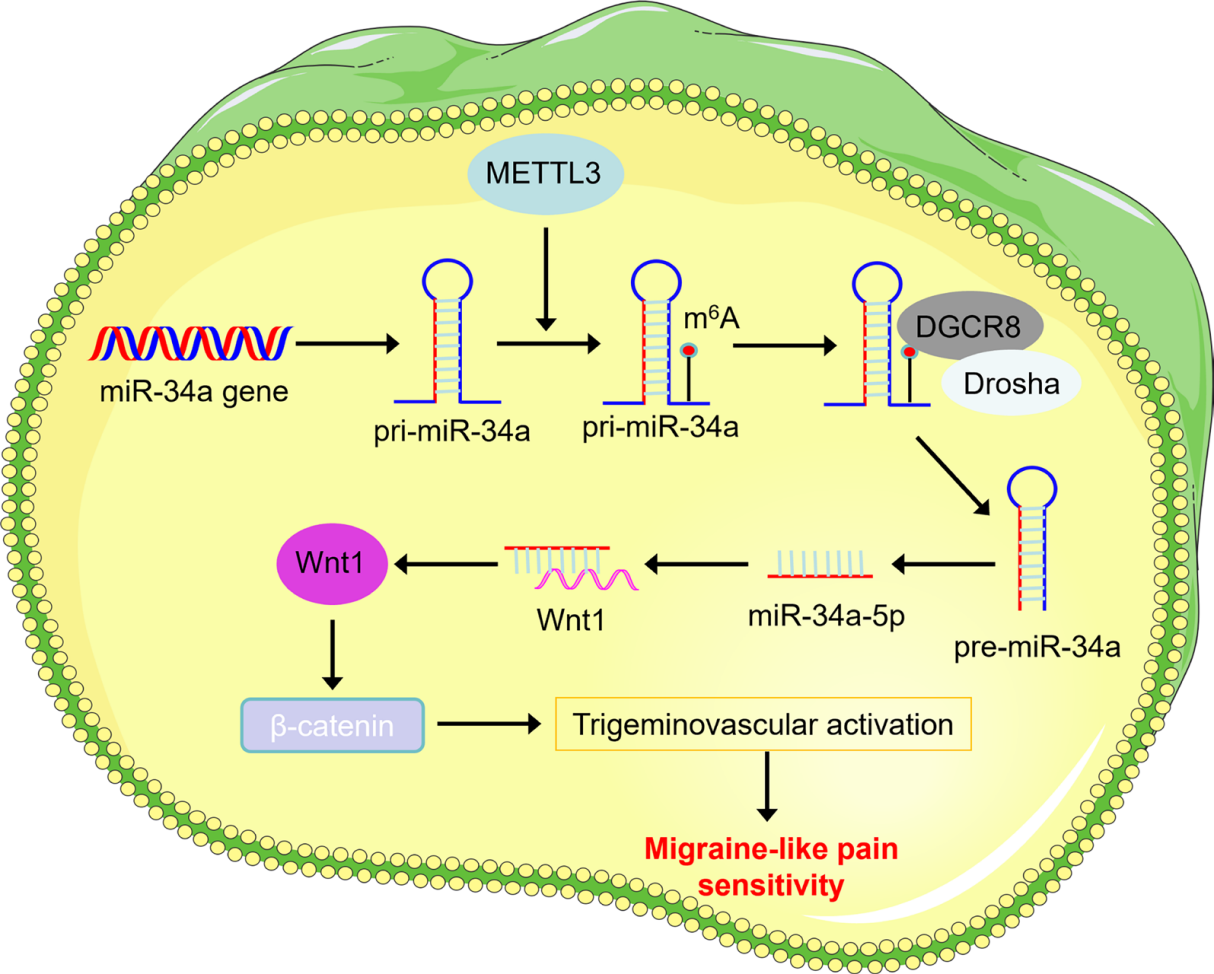
Schematic diagram of METTL3 modulating the activation of the TGVS in migraine. METTL3 enhances the recognition of pri-miR-34a by DGCR8 through m^6^A modification, subsequently processing it into mature miR-34a-5p. miR-34a-5p activates the TGVS and mediates migraine by targeting the Wnt1/β-catenin pathway

Our findings elucidate a novel epigenetic cascade in migraine pathogenesis: METTL3-mediated m^6^A modification facilitates DGCR8-dependent processing of pri-miR-34a into mature miR-34a-5p, which subsequently exacerbates the activation of TGVS through the targeted suppression of the Wnt1/β-catenin signaling pathway (Fig. 9). This mechanistic axis not only bridges RNA epitranscriptomics with neurovascular dysregulation but also positions METTL3 and miR-34a-5p as potential therapeutic targets for mitigating migraine-associated nociceptive sensitization.

## Supplementary Information

Supplementary Material 1

## Data Availability

Not applicable.

## List of Abbreviations

miRNAs: MicroRNAs
NTG: Nitroglycerin
TG: Trigeminal ganglia
TGVS: Trigeminovascular system
CGRP: Calcitonin gene-related peptide
PACAP: Pituitary adenylate cyclase-activating polypeptide
METTL3: Methyltransferase-like 3
m^6^A: N6-methyladenosine
qRT-PCR: Quantitative real-time polymerase chain reaction
COX2: Cyclooxygenase-2
ELISA: Enzyme-linked immunosorbent assay
Co-IP: Co-immunoprecipitation
RIP: RNA immunoprecipitation
NO: Nitric oxide
ANOVA: Analysis of variance
RIPA: Radioimmunoprecipitation assay
BCA: Bicinchoninic acid

## Data availability

No datasets were generated or analysed during the current study.

## Declarations

### Generative AI and AI-assisted technologies in the writing process

During the preparation of this work the authors did not use any AI-assisted technology.

## References

[1] Dong L, Dong W, Jin Y, Jiang Y, Li Z, Yu D (2025) The global burden of migraine: a 30-year trend review and future projections by age, sex, country, and region. Pain Ther 14(1):297–315 10.1007/s40122-024-00690-7 39661241 10.1007/s40122-024-00690-7 PMC11751287

[2] Lyu S, Zhang CS, Sun J et al (2022) Chinese herbal medicine for migraine management: a hospital-based retrospective analysis of electronic medical records. Front Med (Lausanne) 9:936234 10.3389/fmed.2022.936234 10.3389/fmed.2022.936234 PMC9684313 36438031

[3] Wang M, Tutt JO, Dorricott NO, Parker KL, Russo AF, Sowers LP (2022) Involvement of the cerebellum in migraine. Front Syst Neurosci 16:984406 10.3389/fnsys.2022.984406 36313527 10.3389/fnsys.2022.984406 PMC9608746

[4] Goadsby PJ, Holland PR, Martins-Oliveira M, Hoffmann J, Schankin C, Akerman S (2017) Pathophysiology of migraine: a disorder of sensory processing. Physiol Rev 97(2):553–622. 10.1152/physrev.00034.2015 28179394 10.1152/physrev.00034.2015 PMC5539409

[5] Li M, Huang S, Li J, Hu X, Chen J (2025) Health technology assessment: evaluation of 8 CGRP-targeted therapy drugs for the treatment of migraine. Drug Des Devel Ther19:1231–1247 10.2147/DDDT.S499848 39991088 10.2147/DDDT.S499848 PMC11847418

[6] Boinpally R, Shebley M, Trugman JM (2024) Atogepant: mechanism of action, clinical and translational science. Clin Transl Sci 17(1):e13707 10.1111/cts.13707 38266063 10.1111/cts.13707 PMC10777605

[7] Rohatgi S, Gundewar S, Nirhale S et al (2024) The intersection of migraine and epistaxis: clinical observations and analysis. Cureus 16(7):e65584. 10.7759/cureus.65584 39192906 10.7759/cureus.65584 PMC11349249

[8] Turkel CC, Aurora S, Diener HC et al (2023) Treatment of chronic migraine with botox (onabotulinumtoxinA): development, insights, and impact. Medicine (Baltimore) 102(S1):e32600 10.1097/MD.0000000000032600 37499085 10.1097/MD.0000000000032600 PMC10374186

[9] Wang SJ, Chen PK, Fuh JL (2010) Comorbidities of migraine. Front Neurol 1:16 10.3389/fneur.2010.00016 21188255 10.3389/fneur.2010.00016 PMC3008936

[10] Ankrom W, Bondiskey P, Li CC et al (2020) Ubrogepant is not associated with clinically meaningful elevations of alanine aminotransferase in healthy adult males. Clin Transl Sci 13(3):462–472 10.1111/cts.12728 31899602 10.1111/cts.12728 PMC7214647

[11] Romero-Reyes M, Uyanik JM (2014) Orofacial pain management: current perspectives. J Pain Res 7:99–115. 10.2147/JPR.S37593 24591846 10.2147/JPR.S37593 PMC3937250

[12] Sun YY, Zhang WJ, Dong CL et al (2017) Baicalin alleviates nitroglycerin-induced migraine in rats via the trigeminovascular system. Phytother Res 31(6):899–905 10.1002/ptr.5811 28488307 10.1002/ptr.5811

[13] Zhao LP, Liu L, Pei P, Qu ZY, Zhu YP, Wang LP (2017) Electroacupuncture at Fengchi (GB20) inhibits calcitonin gene-related peptide expression in the trigeminovascular system of a rat model of migraine. Neural Regen Res 12(5):804–811 10.4103/1673-5374.206652 28616038 10.4103/1673-5374.206652 PMC5461619

[14] Labastida-Ramírez A, Caronna E, Gollion C et al (2023) Mode and site of action of therapies targeting CGRP signaling. J Headache Pain 24(1):125. 10.1186/s10194-023-01644-8 37691118 10.1186/s10194-023-01644-8 PMC10494408

[15] Zhu L, Liu C, Wang Y et al (2025) METTL3/IGF2BP2/IkappaBalpha axis participates in neuroinflammation in alzheimer’s disease by regulating M1/M2 polarization of microglia. Neurochem Int 186:105964. 10.1016/j.neuint.2025.105964 40107503 10.1016/j.neuint.2025.105964

[16] He X, Zhang J, Guo Y, Yang X, Huang Y, Hao D (2023) METTL3-mediated N6-methyladenosine modification of lncRNA D26496 suppresses the proliferation and migration of Schwann cells after sciatic nerve injury. Mol Neurobiol 60(5):2413–2425. 10.1007/s12035-023-03222-0 36656457 10.1007/s12035-023-03222-0

[17] Grodzka O, Słyk S, Domitrz I (2023) The role of microrna in migraine: a systemic literature review. Cell Mol Neurobiol 43(7):3315–3327. 10.1007/s10571-023-01387-9 37432603 10.1007/s10571-023-01387-9 PMC10477106

[18] Zhang H, He SD, Zong DD, Zhang XM, Luo J, Zheng JK (2020) Effects ofelectroacupuncture on miR-34a-5p/SIRT1 signaling in the trigeminal ganglion of ratswith migraine. Zhen Ci Yan Jiu 45(11):868–874. 10.13702/j.1000-0607.200378 33269829 10.13702/j.1000-0607.200378

[19] Zhang H, Zhang XM, Zong DD et al (2021) MiR-34a-5p up-regulates the IL-1β/COX2/PGE2 inflammation pathway and induces the release of CGRP via inhibition of SIRT1 in rat trigeminal ganglion neurons. FEBS Open Bio 11(1):300–311. 10.1002/2211-5463.13027 33155431 10.1002/2211-5463.13027 PMC7780114

[20] Liu W, Jiang T, Zheng W et al (2023) FTO-mediated m6A demethylation of pri-miR-3591 alleviates osteoarthritis progression. Arthritis Res Ther 25(1):53. 10.1186/s13075-023-03035-5 37005694 10.1186/s13075-023-03035-5 PMC10067311

[21] Han J, Wang JZ, Yang X et al (2019) METTL3 promote tumor proliferation of bladder cancer by accelerating pri-miR221/222 maturation in m6A-dependent manner. Mol Cancer 18(1):110. 10.1186/s12943-019-1036-9 31228940 10.1186/s12943-019-1036-9 PMC6588935

[22] Zhang S, Zhao S, Qi Y et al (2022) SPI1-induced downregulation of FTO promotes GBM progression by regulating pri-miR-10a processing in an m6A-dependent manner. Mol Ther Nucleic Acids 27:699–717. 10.1016/j.omtn.2021.12.035 35317283 10.1016/j.omtn.2021.12.035 PMC8905236

[23] Alarcón CR, Goodarzi H, Lee H, Liu X, Tavazoie S, Tavazoie SF (2015) HNRNPA2B1 Is a mediator of m(6)A-dependent nuclear RNA processing events. Cell 162(6):1299–1308. 10.1016/j.cell.2015.08.011 26321680 10.1016/j.cell.2015.08.011 PMC4673968

[24] Tanha HM, International Headache Genetics Consortium, Nyholt DR (2022) Genetic analyses identify Pleiotropy and causality for blood proteins and highlight Wnt/β-catenin signalling in migraine. Nat Commun 13(1):2593. 10.1038/s41467-022-30184-z 35546551 10.1038/s41467-022-30184-z PMC9095680

[25] Zhang L, Zhou Y, Wang Y et al (2023) Inhibiting PAC1 receptor internalization and endosomal ERK pathway activation may ameliorate hyperalgesia in a chronic migraine rat model. Cephalalgia 43(4):3331024231163131. 10.1177/03331024231163131 36946245 10.1177/03331024231163131

[26] Tian R, Zhang Y, Pan Q et al (2022) Calcitonin gene-related peptide receptor antagonist BIBN4096BS regulates synaptic transmission in the vestibular nucleus and improves vestibular function via PKC/ERK/CREB pathway in an experimental chronic migraine rat model. J Headache Pain 23(1):35. 10.1186/s10194-022-01403-1 35260079 10.1186/s10194-022-01403-1 PMC8903578

[27] Hu J, Ji WJ, Liu GY et al (2025) IDO1 modulates pain sensitivity and comorbid anxiety in chronic migraine through microglial activation and synaptic pruning. J Neuroinflammation 22(1):42. 10.1186/s12974-025-03367-w 39966822 10.1186/s12974-025-03367-w PMC11837436

[28] Chaplan SR, Bach FW, Pogrel JW, Chung JM, Yaksh TL (1994) Quantitative assessment of tactile allodynia in the rat paw. J Neurosci Methods 53(1):55–63. 10.1016/0165-0270(94)90144-9 7990513 10.1016/0165-0270(94)90144-9

[29] Fila M, Sobczuk A, Pawlowska E, Blasiak J (2022) Epigenetic connection of the calcitonin gene-related peptide and its potential in migraine. Int J Mol Sci 23(11):6151. 10.3390/ijms23116151 35682830 10.3390/ijms23116151 PMC9181031

[30] Qian Z, Yang J, Shi L et al (2025) Exploring the role of ubiquitination modifications in migraine headaches. Front Immunol 16:1534389. 10.3389/fimmu.2025.1534389 39958329 10.3389/fimmu.2025.1534389 PMC11825825

[31] De Silva C, Da Costa J (2024) Paracentral acute middle maculopathy associated with migraine with aura in pregnancy. Cureus 16(12):e75072. 10.7759/cureus.75072 39634201 10.7759/cureus.75072 PMC11615411

[32] Huang Z, Zhang Y, Wang S et al (2024) FOXD3-mediated transactivation of ALKBH5 promotes neuropathic pain via m6A-dependent stabilization of 5-HT3A mRNA in sensory neurons. Proc Natl Acad Sci U S A 121(6):e2312861121. 10.1073/pnas.2312861121 38285939 10.1073/pnas.2312861121 PMC10861880

[33] Greco R, Demartini C, Francavilla M, Zanaboni AM, Tassorelli C (2022) Antagonism of CGRP receptor: central and peripheral mechanisms and mediators in an animal model of chronic migraine. Cells 11(19):3092. 10.3390/cells11193092 36231054 10.3390/cells11193092 PMC9562879

[34] Greco R, Demartini C, Francavilla M et al (2024) Effects of the dual FAAH/MAGL inhibitor AKU-005 on trigeminal hyperalgesia in male rats. Cells 13(10):830. 10.3390/cells13100830 38786051 10.3390/cells13100830 PMC11119298

[35] Zhang K, Li P, Jia Y, Liu M, Jiang J (2022) Non-coding RNA and n6-methyladenosine modification play crucial roles in neuropathic pain. Front Mol Neurosci 15:1002018. 10.3389/fnmol.2022.1002018 36466810 10.3389/fnmol.2022.1002018 PMC9716653

[36] Wu L, Tang H (2023) The role of N6-methyladenosine modification in rodent models of neuropathic pain: from the mechanism to therapeutic potential. Biomed Pharmacother 166:115398. 10.1016/j.biopha.2023.115398 37647691 10.1016/j.biopha.2023.115398

[37] Zhang L, Zhao X, Wang J et al (2022) METTL3 suppresses neuropathic pain via modulating N6-methyladenosine-dependent primary miR-150 processing. Cell Death Discov 8(1):80. 10.1038/s41420-022-00880-2 35210391 10.1038/s41420-022-00880-2 PMC8873433

[38] Wu L, Ning P, Liang Y et al (2024) Methyltransferase METTL3 regulates neuropathic pain through m6A methylation modification of SOCS1. Neuropharmacology 261:110176. 10.1016/j.neuropharm.2024.110176 39357736 10.1016/j.neuropharm.2024.110176

[39] Su X, Qu Y, Mu D (2023) The regulatory network of METTL3 in the nervous system: diagnostic biomarkers and therapeutic targets. Biomolecules 13(4):664. 10.3390/biom13040664 37189411 10.3390/biom13040664 PMC10135467

[40] Gallelli L, Cione E, Peltrone F et al (2019) Hsa-miR-34a-5p and hsa-miR-375 as biomarkers for monitoring the effects of drug treatment for migraine pain in children and adolescents: a pilot study. J Clin Med 8(7):928. 10.3390/jcm8070928 31252698 10.3390/jcm8070928 PMC6679182

[41] Gao F, Yang Z, Li J (2025) The miR-34a-5p promotes hippocampal neuronal ferroptosis in epilepsy by regulating SIRT1. Neurochem Res 50(2):124. 10.1007/s11064-025-04378-y 40126751 10.1007/s11064-025-04378-y

[42] Diao LT, Xie SJ, Lei H et al (2021) METTL3 regulates skeletal muscle specific MiRNAs at both transcriptional and post-transcriptional levels. Biochem Biophys Res Commun 552:52–58. 10.1016/j.bbrc.2021.03.035 33740664 10.1016/j.bbrc.2021.03.035

[43] Sarvari P, Sarvari P, Ramírez-Díaz I, Mahjoubi F, Rubio K (2022) Advances of epigenetic biomarkers and epigenome editing for early diagnosis in breast cancer. Int J Mol Sci 23(17):9521. 10.3390/ijms23179521 36076918 10.3390/ijms23179521 PMC9455804

[44] Wang Q, Hou J, Zeng S, Wang X, Liang Y, Zhou R (2025) METTL3-mediated m (6)A modification of pri-miRNA-31 promotes hypertrophic scar progression. Acta Biochim Biophys Sin (Shanghai) 57(7):1106–1114. 10.3724/abbs.2025033 40109092 10.3724/abbs.2025033 PMC12368013

[45] Li X, Xiong W, Long X et al (2021) Inhibition of METTL3/m6A/miR126 promotes the migration and invasion of endometrial stromal cells in endometriosis. Biol Reprod 105(5):1221–1233. 10.1093/biolre/ioab152 34382070 10.1093/biolre/ioab152 PMC10308507

[46] Zingale VD, Gugliandolo A, Mazzon E (2021) MiR-155: an important regulator of neuroinflammation. Int J Mol Sci 23(1):90. 10.3390/ijms23010090 35008513 10.3390/ijms23010090 PMC8745074

[47] Sun XH, Song MF, Song HD, Wang YW, Luo MJ, Yin LM (2019) miR–155 mediates inflammatory injury of hippocampal neuronal cells via the activation of microglia. Mol Med Rep 19(4):2627–2635. 10.3892/mmr.2019.9917 30720115 10.3892/mmr.2019.9917 PMC6423572

[48] Kong H, Wang H, Zhuo Z et al (2020) Inhibition of miR-181a-5p reduces astrocyte and microglia activation and oxidative stress by activating SIRT1 in immature rats with epilepsy. Lab Invest 100(9):1223–1237. 10.1038/s41374-020-0444-1 32461588 10.1038/s41374-020-0444-1

[49] Wang J, Li G, Qian GH, Hua JH, Wang YQ (2016) Expression analysis of eight amphioxus genes involved in the Wnt/β-catenin signaling pathway. Dongwuxue Yanjiu 37(3):136–143. 10.13918/j.issn.2095-8137.2016.3.136 27265651 10.13918/j.issn.2095-8137.2016.3.136 PMC4914576

[50] Xie YK, Luo H, Zhang SX et al (2022) GPR177 in A-fiber sensory neurons drives diabetic neuropathic pain via WNT-mediated TRPV1 activation. Sci Transl Med 14(639):eabh2557. 10.1126/scitranslmed.abh2557 35385340 10.1126/scitranslmed.abh2557

[51] Tang SJ (2014) Synaptic activity-regulated Wnt signaling in synaptic plasticity, glial function and chronic pain. CNS Neurol Disord Drug Targets 13(5):737–744. 10.2174/1871527312666131223114457 24365183 10.2174/1871527312666131223114457 PMC5646676

[52] Bishop J, Becerra L, Barmettler G et al (2019) Modulation of brain networks by sumatriptan-naproxen in the inflammatory soup migraine model. Pain 160(9):2161–2171. 10.1097/j.pain.0000000000001583 31033778 10.1097/j.pain.0000000000001583 PMC7193782

[53] Tang Y, Chen Y, Liu R, Li W, Hua B, Bao Y (2022) Wnt signaling pathways: A role in pain processing. Neuromolecular Med 24(3):233–249. 10.1007/s12017-021-08700-z 35067780 10.1007/s12017-021-08700-z PMC9402773

[54] Liu J, Yao L, Chen Y, Wang X, Wang K (2025) METTL3-mediated m6A modification of MT1G inhibits papillary thyroid carcinoma cell growth and metastasis via Wnt/beta-catenin pathway. Tissue Cell 95:102902. 10.1016/j.tice.2025.102902 40198928 10.1016/j.tice.2025.102902

[55] Yin J, Ding F, Cheng Z et al (2023) METTL3-mediated m6A modification of LINC00839 maintains glioma stem cells and radiation resistance by activating Wnt/beta-catenin signaling. Cell Death Dis 14(7):417. 10.1038/s41419-023-05933-7 37438359 10.1038/s41419-023-05933-7 PMC10338500

[56] Ahmad SR, Rosendale N (2022) Sex and gender considerations in episodic migraine. Curr Pain Headache Rep 26(7):505–516. 10.1007/s11916-022-01052-8 35679008 10.1007/s11916-022-01052-8 PMC9325838

[57] Merki-Feld GS, Caveng N, Speiermann G, MacGregor EA (2020) Migraine start, course and features over the cycle of combined hormonal contraceptive users with menstrual migraine - temporal relation to bleeding and hormone withdrawal: a prospective diary-based study. J Headache Pain 21(1):81. 10.1186/s10194-020-01150-1 32580694 10.1186/s10194-020-01150-1 PMC7315546

[58] van Lohuizen R, Paungarttner J, Lampl C, MaassenVanDenBrink A, Al-Hassany L (2023) Considerations for hormonal therapy in migraine patients: a critical review of current practice. Expert Rev Neurother 24(1):1–21. 10.1080/14737175.2023.2296610 38112066 10.1080/14737175.2023.2296610 PMC10791067

[59] Peng S, Li C, He Y, Xue L, Guo X (2025) Regulatory roles of RNA binding proteins in the Hippo pathway. Cell Death Discov 11(1):36. 10.1038/s41420-025-02316-z 39890775 10.1038/s41420-025-02316-z PMC11785755

[60] Li N, Wei X, Dai J, Yang J, Xiong S (2025) METTL3: a multifunctional regulator in diseases. Mol Cell Biochem 480(6):3429–3454. 10.1007/s11010-025-05208-z 39853661 10.1007/s11010-025-05208-z

